# The Influences of Rainfall Intensity and Timing on the Assemblage of Dung Beetles and the Rate of Dung Removal in an Alpine Meadow

**DOI:** 10.3390/biology12121496

**Published:** 2023-12-06

**Authors:** Wenxiao Sun, Wenting Tang, Yashi Wu, Shuaibing He, Xinwei Wu

**Affiliations:** Department of Ecology, College of Life Science, Nanjing University, Nanjing 210023, China; swenxiao430@163.com (W.S.); wttang@whu.edu.cn (W.T.); wuyashi200009@163.com (Y.W.); 15670523502@163.com (S.H.)

**Keywords:** climate change, detritus, detritivore, dung decomposition, Qinghai–Tibet Plateau, rainfall pattern

## Abstract

**Simple Summary:**

Changes in precipitation patterns can significantly alter biological processes and associated ecosystem functions. This study aimed to investigate the impacts of rainfall intensity and timing on the structure of dung beetle assemblage and dung mass loss rate in a Tibetan alpine meadow. The results revealed that exposure to rainfall within a 2 h timeframe, regardless of rainfall intensity, significantly reduced the rate of dung mass loss. However, this decrease was not attributed to the activity of dung beetles. Earlier rainfall tended to decrease species richness of dung beetles, while heavier rainfall significantly decreased beetle abundance. These findings suggest that changes in precipitation patterns can influence both the assemblage of dung beetles and the rate of dung decomposition, but may also decouple their relationship under a certain circumstance. The variability in local biological processes and ecosystem functions deserves greater attention within a global climate change scenario.

**Abstract:**

Changes in precipitation patterns, including rainfall intensity and rainfall timing, have been extensively demonstrated to impact biological processes and associated ecosystem functions. However, less attention has been paid to the effects of rainfall intensity and rainfall timing on the assembly of detritivore communities and the decomposition rate of detritus such as animal dung. In a grazed alpine meadow on the eastern Qinghai–Tibet Plateau, we conducted a manipulative experiment involving two levels of rainfall intensity (heavy rainfall, 1000 mL/5 min; light rainfall, 100 mL/5 min) and five levels of rainfall timing (0, 2, 4, 24, and 48 h after yak dung deposition). The aim was to determine the effects of rainfall intensity, timing, and their interaction on the assemblage of dung beetles and dung removal rate during the early stage (i.e., 96 h after yak dung deposition) of dung decomposition. Light rainfall significantly increased species richness in the treatment of 48 h after dung pats were deposited. Heavy rainfall significantly decreased beetle abundance in both the 0 h and 48 h treatments while light rainfall had no effect on beetle abundance. Dung mass loss was significant lower in the 2 h treatment compared to other treatments regardless of rainfall intensity. The structural equation model further revealed that the species richness of dung beetles and dung mass loss were significantly affected by rainfall timing but not by rainfall intensity. However, no significant relationships were observed between any variables examined. These findings suggest that changes in precipitation patterns can influence both the structure of dung beetles and the rate of dung decomposition but may also decouple their relationship under a certain circumstance. Therefore, it is crucial to pay greater attention to fully understand local variability between the biological processes and ecosystem functions within a global climate change scenario.

## 1. Introduction

Extensive evidence has demonstrated the profound impact of global climate change on biological processes and associated ecosystem functions in both terrestrial and aquatic ecosystems [1,2]. Precipitation patterns, a key driver of global climate change, have already undergone significant changes and are predicted to undergo further alterations in the future [3]. Specifically, there has been a widespread increase in rainfall intensity across various regions worldwide [4]. The pronounced seasonal variation in rainfall timing draws attention to extreme events caused by the uneven distribution of rainfall time and changes in rainfall intervals [5,6]. These changes in rainfall patterns have exerted influences on metabolic processes [7,8], population dynamics [9], species interactions [10], assemblages [11], the distribution ranges of natural plants and animals [12,13], as well as ecosystem functions and services [14]. Nevertheless, compared with other drivers such as climate warming, our comprehensive understanding of the impacts resulting from changes in precipitation patterns remains incomplete, particularly regarding their effects on the assemblage of detritivore community and the decomposition rates of non-living organic matter.

The decomposition of dead organic matter is a biogeochemical process that plays a fundamental role in nutrient, carbon, and energy cycling within and among ecosystems, as well as the exchange between the biosphere and atmosphere [15]. The decomposition rates of dead organic matter are largely influenced by the diversity and population dynamics of detritivores responsible for breaking down non-living organic matter into simpler forms to gain energy and matter for their own biomass maintenance [16]. Importantly, the effect sign and strength of these soil micro-, meso- and macro-organisms on decomposition can be modified by rainfall patterns [17]. For instance, a 50% reduction in rainfall significantly decreased the decomposition rate of leaf litter through influencing the activities of soil microorganisms and soil animals [18]. A microcosm experiment investigating leaf litter decomposition revealed that microbial-driven decomposition was positively correlated with cumulative rainfall quantity, while isopod-driven decomposition was strongly controlled by rainfall frequency [19]. However, it should be noted that these studies have primarily focused on the decomposition of plant detritus rather than animal-based materials such as feces or carcasses.

Ignoring the decomposition of animal detritus would be inappropriate [20]. For example, terrestrial plants annually produce approximately 120 billion tons of organic carbon [21], with up to 90% entering the detritus pool as plant litter [22]. Even of the remaining 10% consumed by herbivores, only very little is assimilated for growth and development, while the majority eventually returns to the detritus pool as excreta and carrion [23]. Although these animal detritus components constitute only a small percentage compared to plant detritus within the total natural detritus pool [24,25], they display a disproportionate role in assembling and ecosystem functioning [26]. Firstly, animal detritus provides energy and habitat for a suite of highly dependent species [27]. For example, the whole life history of a variety of dung beetle species depends completely on excreta from large animals. Secondly, compared to plant detritus, animal detritus is an ephemeral resource but very rich in nutrients. The nitrogen content of dung is two to three times higher than that of plant litter [18,28]. Moreover, the rates of nutrient turnover and organic matter decomposition of animal detritus are undoubtedly quicker than plant detritus [24]. This energy pulse of animal detritus is crucial to the maintenance of biodiversity and the normal functioning of many ecosystems.

Like the decomposition of plant detritus, the decomposition of animal detritus also depends largely on the population dynamics, species diversity, and community structure of detritivores [15,29], all of which are sensitive to environmental change [30]. Changes in rainfall patterns are suggested to influence decomposition by modifying physiological traits, feeding behaviors, and species interactions among decomposing arthropods [31,32,33,34]. For example, decreased rainfall significantly reduces the abundance of dung beetles, leading to a decline in dung decomposition rates [32]. Although the onset of rainfall has been linked to the emergence of dung beetle populations for many years, available field data about the effect of the changes in rainfall patterns on the richness, abundance, and ecological functions of dung beetle species are scarce [31,32,33,34]. Given the great substantial variability in global rainfall patterns, further research is necessary for a comprehensive understanding of the relationship between changes in rainfall patterns and animal detritus decomposition.

As the “Third Pole of the Earth”, the Qinghai–Tibet Plateau is a biodiversity hotspot and is particularly sensitive to global climate change [35,36]. Specifically, there has been an increase in rainfall amounts and intensity, frequency of heavy rainy events, and the daily maximum of rainfall during the period from 1961 to 2017 [37]. Additionally, precipitation duration has decreased since 2000 [37], with this trend predicted to persist until the end of the century [38]. Moreover, the Qinghai–Tibet Plateau houses China’s largest area of grassland and rangeland, which supports billions of wild animals and livestock, including yaks (*Bos grunniens* Linnaeus, 1766), which are predominantly reared by local people. The estimated density of yak dung pats can reach up to 5900 per hectare, covering more than 20% area of the total rangeland [39]. The timely decomposition of yak dung is thus important to nutrient turnover and primary productivity. Dung beetles (Coleoptera: Scarabaeoidea) are the major group that contributes substantially to dung decomposition on the Qinghai–Tibet Plateau. Notably, because the species richness and abundance of dung beetles always peak within the first two to three days after dung deposition [39], the early stage of dung decomposition is the best period to examine the population dynamics and assemblage structure of dung beetles. Rainfall events might affect the assemblage and dung decomposition rates by influencing volatile substances released from dung as well as dung beetle behavior. Given the importance of rainfall patterns on detritivores and detritivores on decomposition, we hypothesize here that changes in rainfall patterns will alter decomposition rates through affecting detritivore community assemblage. Specifically, we aim to test whether high-intensity rainfall events that occurred earlier would lead to reduced richness and abundance of dung beetles, which in turn would decrease dung decomposition rates.

## 2. Materials and Methods

### 2.1. Study Site

The experiment was conducted in an alpine meadow in Hongyuan County (32°48′ N, 102°33′ E) in the northwest of Sichuan Province, located in the eastern edge of the Qinghai–Tibet Plateau, China. The average altitude is about 3500 m. The climate is characterized by long, cold winters and short, cool summers. According to the Hongyuan County Climate Station (a 4 km distance to our study site) from 1961 to 2019, the mean annual temperature was 1.7 °C, with the maximum and minimum monthly means being 13.4 °C and −15.1 °C in July and January, respectively. Precipitation mostly (>80%) occurred from May to August. The mean annual precipitation was about 760 mm with significant fluctuations across years, mostly ranging from 450 mm to 900 mm [40]. Details on the species composition of plant community at the study site can be found in previous studies [39]. The soil is characterized by high organic content and low nitrogen and phosphorus content. The meadow has been under intensive grazing (by yaks) for decades. The study site was a typical summer-grazing pasture, which was under grazing from May to September by local people. According to an independent pre-experimental survey, the estimated population density is about 10 yaks per hectare.

The decomposer community responsible for yak dung decomposition in the Tibetan alpine meadows is as complicated as in other areas [41]. In addition to the microfauna (bacteria and fungi), macro-invertebrates such as beetles and flies are dominant decomposer groups especially in the early stages of dung decomposition. In our study area, *Calliphora vicina* (Robineau-Desvoidy, 1830) is usually the most abundant fly species. The coprophagous beetle species include *Eupleurus subterraneus* (Linnaeus, 1758), *Aphodius erraticus* (Linnaeus, 1758), *A. rectus* (Motschulsky, 1866), *A. prodromus* (Brahm,1790), *A. elegans* (Allibert, 1847), *Geotrupes ibericus* (Baraud, 1958), *Onthophagus uniformis* (Heyden, 1886), etc. Predaceous beetles include *Philonthus rubripennis* (Stephens, 1832), *Quedius (Microsaurus) liangshanensis* (Zheng and Zheng, 2006), and *Sphaerites* sp. In the very late successional stage, there may be spiders (e.g., *Araneus marmoreus* (Clerk, 1757)), centipedes, and beetle larvae emerging within and underneath the dung [42].

### 2.2. Experimental Design and Sampling

The experiment was conducted using a two-factor full design, where rainfall intensity with two levels (heavy rainfall, 1000 mL rainwater in five mins; light rainfall, 100 mL rainwater in five mins), and rainfall timing with five levels (0, 2, 4, 24 and 48 h after fresh dung pats were deposited). The heavy rainfall was a little beyond the usual maximum rainfall. Notably, however, the classification of the heavy and light rainfall here was not a meteorological definition. There were thus ten treatments, each with five replicates, and each replicate was an artificial fresh dung pat. There were 50 dung pats in total.

The experiment was conducted in a flat pasture with an area of about two hectares where the vegetation cover is over 80%. Before the start of the experiment, enough rainwater was collected and stored in buckets. In the early morning of a sunny day (20 August 2021), enough fresh yak dung free of any macro-invertebrates was collected in a stall of a Tibet family. After thoroughly mixing in large buckets, the dung was built into small artificial pats using plastic modules with a diameter of 18 cm and a height of 4 cm, respectively. The fresh weight of each pat was 1000 ± 20 g (250 ± 5 g in dry weight). The pats were then randomly placed in the pasture, with at least an interval of 5 m between any two pats to reduce possible mutual interference with each other. Finally, rainwater was sprayed evenly on the surface of each pat using watering pots according to the experimental design. In addition, two additional pats were arranged as candidate compensates for each treatment. Pats damaged by grazing yaks or rodents during the experimental period would have been randomly replaced by these candidates. In addition, the temperature was measured using thermometers (model DS1921G, Maxim Integrated Products, Sunnyvale, CA, USA). Over the course of the experiment, the mean was 12.21 °C based on daily average (over 24 h of measurements made every 30 min), with the highest and lowest values being 28.5 °C and 0 °C, respectively.

On the fourth day (i.e., 96 h after deposition of yak dung) since the beginning of the experiment, dung beetles and any other visible decomposers (if any) within both the dung pats and the soil beneath the pats were collected by hand-sorting and counted. Dung beetles were identified to the species level. Furthermore, the residual dung was also collected and dried at 75 °C for 72 h to assess the rate of dung mass loss. It is important to note that there was no rainfall throughout the entire experimental period.

### 2.3. Data Analysis

Two-way ANOVA was used to test the effects of rainfall intensity, rainfall timing, and their interaction on dung mass loss, species richness and abundance (log-transformed) of dung beetles. Once a significant factor effect was detected, post hoc Tukey’s HSD tests were used to further determine the differences among treatments. The relative density of each species was calculated by dividing the abundance of the specie in the treatment by the total abundance of all beetles in the treatment. Then, the similarity percentage (SIMPER) analysis based on the Bray–Curtis similarity matrix was employed to determine the species contribution among different treatments [40]. In addition, a piecewise structural equation model (SEM) was constructed to examine the causal relations among rainfall, dung beetles, and dung mass loss. An initial SEM model was constructed based on our hypothesis that dung decomposition would be affected either directly by rainfall patterns or indirectly by rainfall patterns through affecting dung beetle assemblages (Figure S1). In doing this, collinearity between variables in the model was firstly assessed through a variance inflation factor (VIF) test using the car package [43]. Because our terms had more than 1 degree of freedom (df), generalized variance-inflation factors [44] were calculated. Secondly, the model was performed with the piecewiseSEM package using the maximum likelihood estimation method [45]. The conceptual model was evaluated based on goodness-of-fit statistics and the selection of optimal model relied on the lowest AIC value. In the model, both main and interactive effects of rainfall intensity and timing were considered. All these analyses were performed in R 4.1.1 [46].

## 3. Results

### 3.1. Responses of Beetle Assemblage and Dung Decomposition

**Figure 1:**
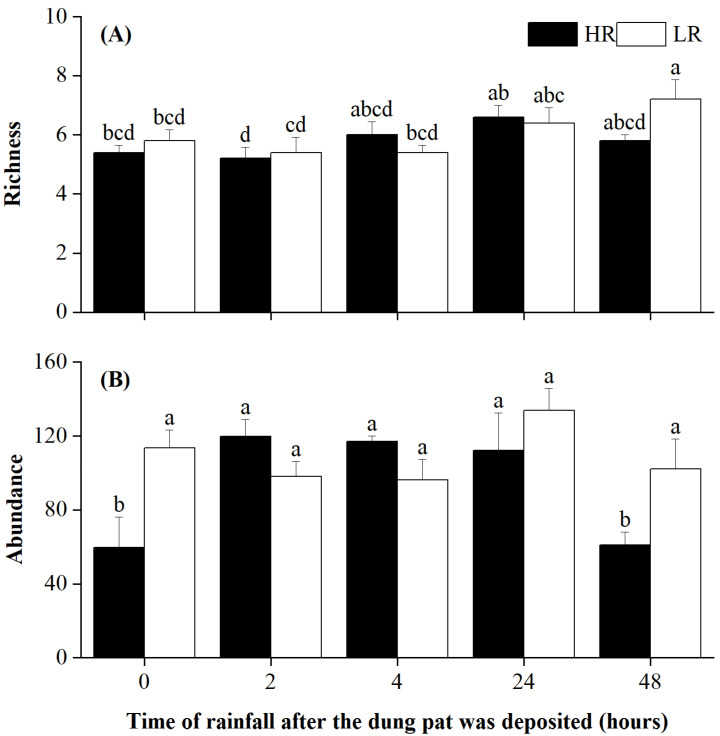
Species richness (**A**) and abundance (**B**) of dung beetles in different treatments at the end of the experiment. The letters above the error bars denote statistically significant differences among treatments at a significant level of *p* = 0.05, as revealed by two-way ANOVAs followed by post hoc Tukey’s test for multiple comparisons. Error bars are ±SE.

Rainfall timing significantly affected the species richness and abundance of dung beetles (Table 1). Rainfall intensity and its interaction with rainfall timing significantly affected beetle abundance (Table 1). Specifically, light rainfall led to a significant increase in species richness in the 48 h treatment (Figure 1A). On the other hand, heavy rainfall resulted in a significant decrease in beetle abundance for both the 0 h treatment and the 48 h treatment, while light rainfall had no effect on beetle abundance (Figure 1B).

**Figure 2:**
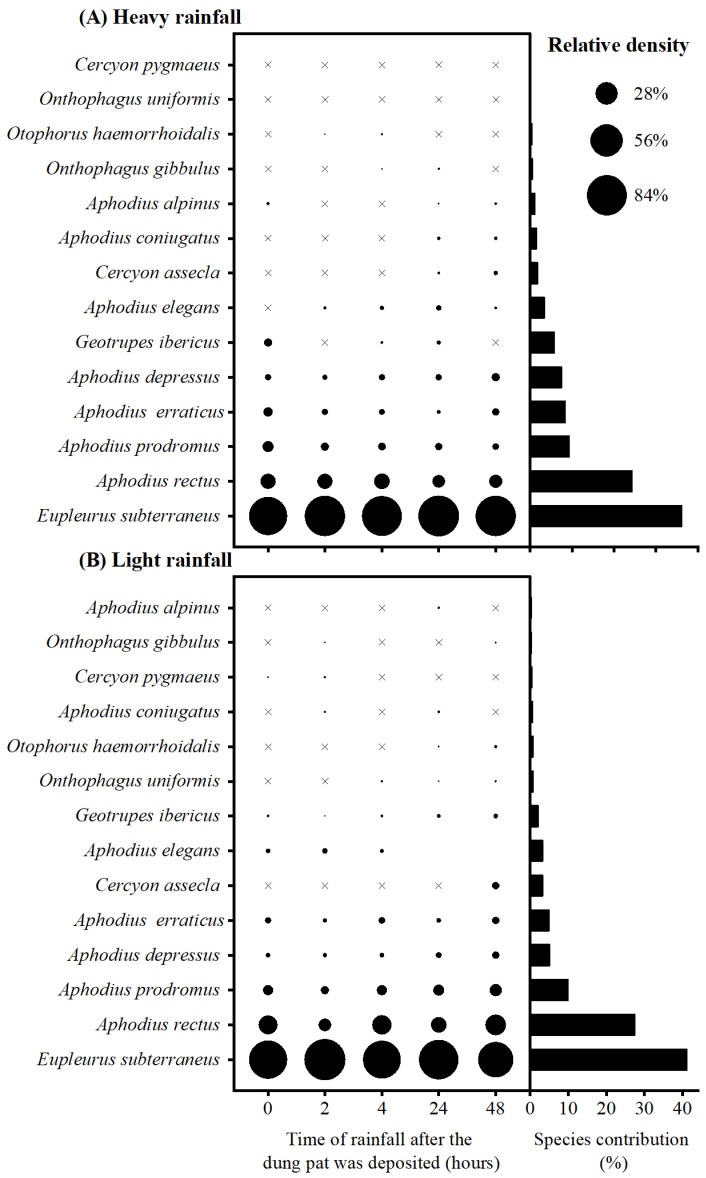
The relative density of different species in different treatments, and species with their corresponding contributions in SIMPER analysis of heavy rainfall (**A**) and light rainfall (**B**). “×” means the relative density of the specie is zero in the treatment.

A total of 5067 dung beetle individuals were collected, belonging to three families and 14 species (Table S1). The average dissimilarity among different rainfall timings were 13.62% and 16.07% in the heavy rainfall and light rainfall conditions, respectively. In the heavy rainfall conditions, the dissimilarity was primarily driven by the contribution of seven species (>2%), which accounted for a significant proportion (95%) of the overall dissimilarity. Notably, *E. subterraneus* contributed approximately 36% to the dissimilarity (Figure 2A). Similarly, in the light rainfall conditions, the dissimilarity was mainly influenced by the presence of eight species that collectively accounted for 96.85% of the overall dissimilarity. Among these species, *E. subterraneus* displayed a prominent role with an approximate contribution of about 41% (Figure 2B). Furthermore, it is worth mentioning that there were no evident differences in relative density of *E. subterraneus* across different treatments (Figure 2).

**Figure 3:**
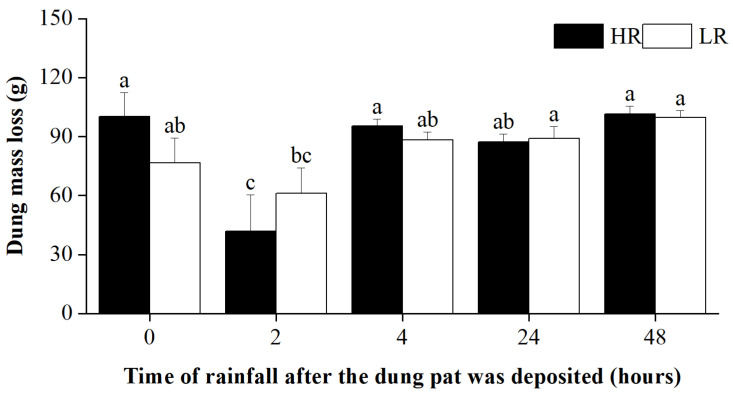
Dung mass loss in different treatments at the end of the experiment. The letters above the error bars denote statistically significant differences among treatments at a significant level of *p* = 0.05, as revealed by two-way ANOVAs followed by post hoc Tukey’s test for multiple comparisons. Error bars are ±SE.

In addition, rainfall timing significantly affected dung mass loss (Table 1). The dung mass loss was lower in the 2 h treatment compared to other treatments regardless of rainfall intensity (Figure 3).

### 3.2. Relationships among Rainfall, Beetle Assemblage, and Dung Decomposition

**Figure 4:**
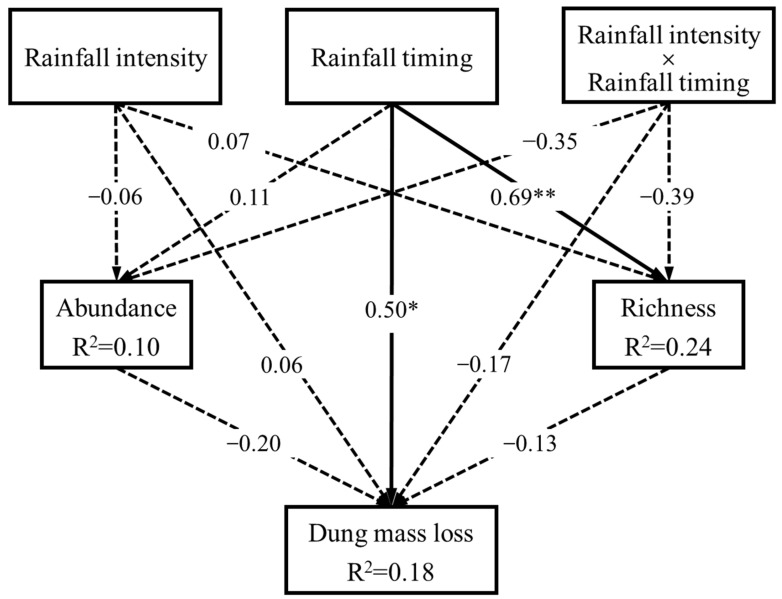
Structural equation model exploring the casual relationship among rainfall, dung beetles, and dung mass loss (Fisher’ C = 2.91, *p* = 0.23, AIC = 36.91; a low AIC value associated with Fisher’s C test indicates a strong fit of the model to data). Arrows represent unidirectional relationships between variables, with standardized path coefficients (β) indicated on each arrow. Solid arrows represent significant relationships, while dashed arrows represent non-significant relationships. **, *p* < 0.01; *, *p* < 0.05.

The results of the SEM showed that species richness of dung beetles (β = 0.69) as well as the dung mass loss (β = 0.50) were significantly and positively influenced by rainfall timing rather than rainfall intensity or their interaction. However, no significant relationships were observed between any other variables (Figure 4).

## 4. Discussion

Compared to plant litter, the organic matter in animal dung generally decomposes at a faster rate due to the presence of a more diverse and abundant decomposer community [28]. Nevertheless, it is evident that the composition of dung decomposers varies significantly among different regions or ecosystems. For example, earthworms play a primary role in degrading cattle dung in temperate climatic areas [47,48], while termites contribute significantly in arid [49] or seasonally dry areas [50]. In tropical, temperate and highland grassland ecosystems, dung beetles are typically the most important dung-feeding guild responsible for dung decomposition and nutrient cycling [39,51]. Although maggots have also been reported to be one important dung decomposer group in the Tibetan alpine grasslands [39], it is curious that we did not observe their presence within dung pats during our experimental period. We speculate that this might be due to the low temperature, which could have limited the flying and egg-laying as well as egg-hatching activities of flies during the experiment. Therefore, we here focused primarily on the responses of dung beetles. Previous studies have revealed that changes in environmental conditions can influence the community structure of dung beetles and subsequently impact dung decomposition rates [32,51]. However, we did not find any clear evidence supporting our hypothesis that rainfall influenced dung decomposition rates by altering the structure of dung beetles. Indeed, we found that both rainfall intensity and rainfall timing significantly affected the structure of dung beetles, and also a significant effect of rainfall timing on the rate of dung mass loss. However, changes in rainfall patterns simultaneously decoupled the links between dung beetles and dung decomposition. These results validate the fact that local-scale environmental changes can alter the inherent relationships between biological processes and associated ecosystem functions.

Our results showed that rainfall timing significantly influenced the species richness of dung beetles, that is to say, the rain played an important role in determining whether beetles visited a fresh dung pat successfully. The species composition of the beetle assemblage within dung pats usually exhibits a distinct successional pattern during the early stage of dung decomposition [41], which is believed to be closely linked to the emission and component of volatile organic compounds (VOCs) from fresh dung pats [52]. These VOCs, driven by microbes, provide dung beetles with multifaceted information including the location, type, and condition of the potential resources [53]. Therefore, any changes in environments that disrupt the emission of VOCs may disturb the selection by and visitation of dung beetles of their target resource. In our study, it can be inferred that rainfall might have affected the normal release of volatiles from dung pats [54], thereby impeding dung beetle’s ability to locate target dung pats and reducing the species diversity of dung beetles. Moreover, it appears that a later (e.g., 48 h) rainfall had a weaker effect on the species richness. This could largely be attributed to the crust formed on the surface of dung pat which may have offset part of the rainfall effect. Interestingly, rainfall intensity did not have a significant effect on the species richness of dung beetles. Nevertheless, heavy rainfall, particularly when it occurred earlier (i.e., 0 h after pats were deposited), resulted in a significant decline in beetle abundance. This could potentially be because that a heavy rainfall might have made the surface of dung pats viscous enough for beetles to crawl into. Once the crust was successfully formed, the impact of heavy rainfall on beetle abundance became negligible as observed in the 2-h, 4 h and 24 h treatments. In addition, the flight of beetles may have been hindered by heavy rainfall, particularly during an actual rainfall event. It is curiously that beetle abundance in the 48 h treatment was observed to decrease under the heavy rainfall condition. We speculate that this could be attributed to the higher water content within dung pats induced by heavy rainfall, which exerted stress on dung beetles. Notably, the species *E. subterraneus* always predominated the beetle assemblage regardless of changes in rainfall patterns. This probably suggests that this particular species may have a low sensitivity to rainfall events. In this end, we boldly speculate that with regard to rainfall patterns, rainfall timing can partly determine the number of beetle species attracted to a dung pat, while rainfall intensity determines the number of beetle individuals can dwell within a pat.

Given the important role of dung beetles in the decomposition process of dung, changes in species diversity and the abundance of dung beetles should lead to corresponding responses in dung decomposition rates. However, our findings indicate that there was no significant correlation between the pattern of dung mass loss and the richness and abundance of dung beetles. This finding was inconsistent with results reported in many other studies [32,55]. This may be because we cannot separate the rainfall effect from the beetle effect due to our experimental design. Differences in the geographic location may also have an important influence. Indeed, rainfall timing was observed to significantly impact on dung mass loss, i.e., dung mass loss was significantly lower when fresh dung pats that were deposited two hours later, regardless of rainfall intensity. In case of the fresh dung pats (e.g., pats in the 0 h treatment), their compact surface may prevent rainfall from penetrating into the pats. However, as the dung pat aged, the crust on its surface, liquid within it, along with holes created by beetles, may have collectively facilitated a leaching effect [56]. Notably, however, once the dung pat became drier (e.g., after depositing for 4 h), the leaching effect might become negligible.

As a special type of natural resource, dung from large vertebrates has been recognized as an important ecological unit for its high biodiversity, intriguing successional process, and important role in ecosystem functioning [41]. In particular, the status of dung pats during the early stage (i.e., the first two to three days after dung is dropped) of dung decomposition can largely determine the community structure of dung decomposers, which in turn influences the rates of organic matter decomposition and nutrient release. Several deficiencies and specialties of our experiment need to be clarified. Firstly, it should be noted that the present study employed a manipulated experiment to simulate rain conditions on a specific resource, which deviated from natural rainfall patterns occurring over a broad geographical region. This disparity may elicit distinct effects on dung beetles. For instance, while the humidity level of the resource might not necessarily impact the flight activity of beetles, intense precipitation can have an effect. Secondly, the observed responses of beetle adults and dung decomposition rate may not fully capture the overall effects resulted from changes in rainfall patterns on dung decomposition because this study solely focused on the early stage of dung decomposition processes, and it was lacking a real experiment control. Actually, the complete breakdown of organic matter and nutrient release from dung requires an extended period spanning several months or even 1 to 2 years on the Qinghai–Tibet Plateau [39]. In particular, dung beetle larvae contribute largely to the loss of organic matter during the middle and late stages of decomposition. Thirdly, the species composition and diversity of decomposers was simplistic, reflecting the decomposer community on the Qinghai–Tibet Plateau. For example, the species *E. subterraneus* occupied for more than 80% of the total beetle abundance. The experiment was conducted in the late August, a period characterized by a relatively low temperature, which could result in the absence of some decomposer groups such as flies and certain species of dung beetles. Furthermore, this study was carried out in a geographically specific climatic area particularly characterized by high amounts and a strong intensity of precipitation. Therefore, the results should be cautiously generalized to other areas. In this end, considering the increasing variability in rainfall patterns on the Qinghai–Tibet Plateau [37,38] as well as other grazing ecosystems, more local research is urgently needed to fully comprehend the relationship between the biological processes and detritus decomposition.

## 5. Conclusions

In this study, we revealed that rainfall patterns, particularly rainfall timing, exerted a significantly impact on the structure of dung beetles and dung mass loss rate; meanwhile, the relationship between dung beetles and dung mass loss appeared to be decoupled in a Tibetan alpine meadow. These findings provide clear evidence that changes in precipitation patterns can influence the correlation between the species diversity of dung beetles and the decomposition rate of yak dung. This may contribute to our understanding of the relationship between biodiversity–ecosystem function regarding detrital systems under the environmental change conditions.

## Figures and Tables

**Table 1:** Results of a two-way ANOVA showing the effects of rainfall timing (timing), rainfall intensity (intensity), and their interaction on the species richness, abundance of dung beetles, and dung mass loss.

	df	Richness		Abundance		Dung Mass Loss	
		SS	F	SS	F	SS	F
Timing	4	0.06	3.43 *	0.35	3.83 **	14,350	7.70 ***
Intensity	1	0.00	0.55	0.12	5.37 *	61	0.13
Timing × Intensity	4	0.02	1.31	0.38	4.27 **	2401	1.29
Residuals	40	0.18		0.90		18,627	

*, *p* < 0.05; **, *p* < 0.01, ***, *p* < 0.001.

## Data Availability

The data presented in this study are available upon request from the corresponding author.

## Supplementary Materials

The following supporting information can be downloaded at: https://www.mdpi.com/article/10.3390/biology12121496/s1, Table S1: The number of different beetle species in different treatments during the experiment period; Figure S1: Diagram of possible pathways that rainfall patterns and dung beetles affect the dung mass loss.
