# Supplementary material for: The Influences of Rainfall Intensity and Timing on the Assemblage of Dung Beetles and the Rate of Dung Removal in an Alpine Meadow
Source: Biology (Basel). 2023 Dec 6;12(12):1496. doi: 10.3390/biology12121496 (PMC10741044; doi:10.3390/biology12121496)
Supplement: Supplementary file 1 [file biology-12-01496-s001.zip › biology-2621402-supplementary.pdf]

Supplementary Materials for

The influences of rainfall intensity and timing on the assemblage of dung beetles and the rate of dung removal in an alpine meadow

Wenxiao Sun [1], Wenting Tang [1], Yashi Wu [1], Shuaibing He [1], Xinwei Wu [1,*]

[1] Department of Ecology, College of Life Science, Nanjing University, Nanjing 210023, China
* Correspondence: wuxw@nju.edu.cn; Tel.: 86-25-89681213

Table S1. The number of different beetle species in different treatments during the experiment period.

Family	Species	Heavy rainfall					Light rainfall				
		**0**	**2**	**4**	**24**	**48**	**0**	**2**	**4**	**24**	**48**
Scarabaeidae	*Eupleurus subterraneus*	227	490	469	473	249	419	416	348	515	321
	Aphodius rectus	30	65	69	45	27	97	40	88	82	104
	Aphodius prodromus	16	19	16	14	7	27	16	25	40	37
	Aphodius erraticus	12	12	10	5	7	10	4	10	8	13
	Aphodius depressus	6	8	12	9	9	6	4	5	12	15
	Aphodius alpinus	2	0	0	1	1	0	0	0	1	0
	Aphodius elegans	0	3	5	7	1	6	7	3	2	2
	Otophorus haemorrhoidalis	0	1	1	0	0	0	0	0	1	2
	Onthophagus gibbulus	0	0	1	1	0	0	1	0	0	1
	Aphodius coniugatus	0	0	0	2	2	0	1	0	2	0
	Onthophagus uniformis	0	0	0	0	0	0	0	1	1	1
Geotrupidae	*Geotrupes ibericus*	5	0	2	3	0	2	1	1	5	5
Hydrophilide	*Cercyon assecla*	0	0	0	1	2	0	0	0	0	10
	Cercyon pygmaeus	0	0	0	0	0	1	1	0	0	0

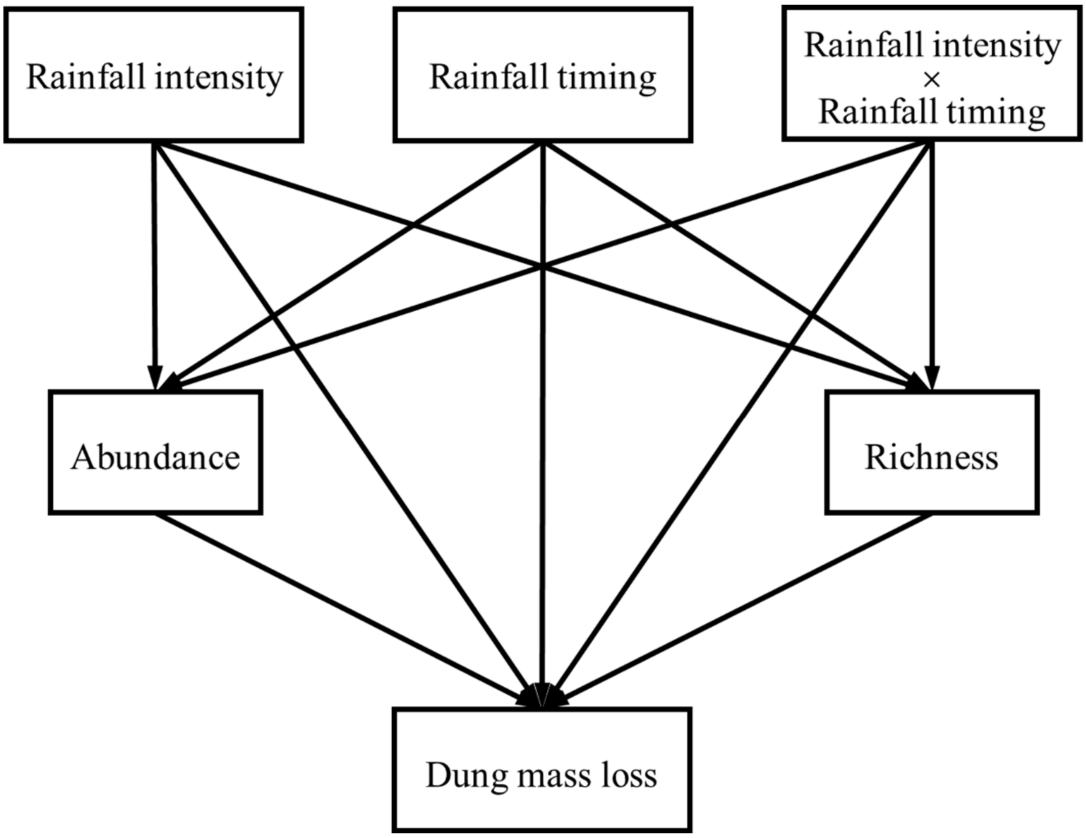

Figure S1. Diagram of possible pathways that rainfall patterns and dung beetles affect the dung mass loss. Rainfall patterns including rainfall intensity, timing and their interaction may directly affect the dung beetle assemblage (abundance and richness) and dung mass loss, and may also affect the dung mass loss by affecting the dung beetle assemblage. Dung beetle assemblage may also directly affect dung mass loss.
